# TMEM106B, a frontotemporal lobar dementia (FTLD) modifier, associates with FTD-3-linked CHMP2B, a complex of ESCRT-III

**DOI:** 10.1186/s13041-015-0177-z

**Published:** 2015-12-10

**Authors:** Mi-Hee Jun, Jeong-Ho Han, Yu-Kyung Lee, Deok-Jin Jang, Bong-Kiun Kaang, Jin-A Lee

**Affiliations:** Department of Biotechnology and Biological Sciences, Hannam University, 1646 Yuseongdaero, Yu-seong-gu, Daejeon 305-811 South Korea; Applied Biology, College of Ecological Environment, Kyungpook National University, 386, Gajang-dong, Sang-Ju, South Korea; Department of Biological Sciences, College of Natural Sciences, Seoul National University, 1 Gwanangno, Gwanak-gu, Seoul 08826 South Korea

**Keywords:** TMEM106B, ESCRT, CHMP2B, FTLD, EGFR, autophagy

## Abstract

**Background:**

Transmembrane protein 106B (TMEM106B) has been identified as a risk factor for frontotemporal lobar degeneration, which is the second most common form of progressive dementia in people under 65 years of age. Mutations in charged multivesicular body protein 2B (CHMP2B), which is involved in endosomal protein trafficking, have been found in chromosome 3-linked frontotemporal dementia. Despite the number of studies on both CHMP2B and TMEM106B in the endolysosomal pathway, little is known about the relationship between CHMP2B and TMEM106B in the endosomal/autophagy pathway.

**Results:**

This study found that endogenous TMEM106B was partially sequestered in CHMP2B-positive structures, suggesting its possible involvement in endosomal sorting complexes required for transport (ESCRT)-associated pathways. The role of single nucleotide polymorphisms of TMEM106B (T185, S185, or S134N) in the ESCRT-associated pathways were characterized. The T185 and S185 variants were more localized to Rab5-/Rab7-positive endosomes compared with S134N, while all of the variants were more localized to Rab7-positive endosomes compared to Rab5-positive endosomes. T185 was more associated with CHMP2B compared to S185. Autophagic flux was slightly reduced in the T185-expressing cells compared to the control or S185-expressing cells. Moreover, T185 slightly enhanced the accumulation of EGFR, impairments in autophagic flux, and neurotoxicity that were caused by CHMP2B^Intron5^ compared to S185-expressing cells.

**Conclusions:**

These findings suggest that the T185 variant functions as a risk factor in neurodegeneration with endolysosomal defects. This study provides a better understanding of pathogenic functions of TMEM106B, which is a risk factor for the progression of neurodegenerative diseases that are associated with endosomal defects in the aged brain.

**Electronic supplementary material:**

The online version of this article (doi:10.1186/s13041-015-0177-z) contains supplementary material, which is available to authorized users.

## Introduction

Frontotemporal lobar degeneration (FTLD) is the third most common neurodegenerative disease, after Alzheimer’s disease and Parkinson’s disease [1]. FTLD is a genetically, clinically, and pathologically heterogeneous disease with symptoms that include progressive aphasia and changes in personality and social behavior. Interestingly, about 40 % of patients with FTLD show a family history of the disease, which indicates that FTLD has a strong genetic component [2]. Indeed, several disease-associated genes, such as microtubule-associated protein tau (*MAPT*)*,* progranulin (*PGRN*)*,* charged multivesicular body protein 2B (*CHMP2B*)*,* chromosome 9 open reading frame 72 (*C9ORF72*), and valosin-containing protein (*VCP*)*,* have been identified as causative or risk factors for FTLD [3, 4]. A recent genome-wide association study of FTLD with TAR DNA-binding protein 43 (TDP-43) inclusions (FTLD-TDP), or FTLD with a *PGRN* mutation, showed that transmembrane protein 106B (TMEM106B), which encodes a transmembrane protein with unknown function, increases the risk of the disease or of the development of cognitive impairment in patients with amyotrophic lateral sclerosis [5, 6]. Furthermore, its expression changes in patients with Alzheimer’s disease [7, 8]. Linkage disequilibrium studies have shown that the top three single-nucleotide polymorphisms (SNPs) (rs6966915, rs1020004, and rs1990622) in the noncoding region of TMEM106B are associated with FTLD-TDP, and TMEM106B mRNA and protein expression are significantly increased in the frontal cortex of patients with FTLD-TDP compared with controls, suggesting its importance in normal brain function [9]. Moreover, the S134N and p.(T185 or S185) variants in the coding region have been identified in patients with FTLD [7]. rs3173615 (p.185S) was found to be in perfect linkage disequilibrium with rs1990622, which is one of the top three SNPs of TMEM106B. This suggests that S185 is a protective isoform while the T185 form confers risk. Protein levels of S185 are reportedly lower than T185 because of its rapid rate of protein degradation in mammalian cells [10]. However, the association of S134N in neurons with disease has not been fully addressed [7]. More recently, TMEM106B has been shown to be a genetic modifier in patients with FTLD with *C9ORF72* expansions, which are the most common known genetic cause of frontotemporal dementia (FTD), amyotrophic lateral sclerosis, and the combination of these diseases [4, 11]. Therefore, TMEM106B is a major genetic modifier in patients with FTLD with a PGRN mutation or *C9ORF72* hexanucleotide repeat expansions [2, 3].

TMEM106B is a type-II glycoprotein localized to late endosomes/lysosomes, and its overexpression causes enlarged lysosomes and impaired endo-lyososomal degradation [12, 13]. The interaction of TMEM106B and MAP6 has been reported to regulate the dendritic trafficking of lysosomes in cultured primary hippocampal neurons, which suggests that TMEM106B plays a crucial role in the regulation of protein trafficking through MAP6 in the dendrites of polarized neurons [14]. More recently, TMEM106B has been shown to regulate lysosome size, motility, and stress responses and interact with endocytic proteins, such as the μ1 subunit of AP2 (AP2M1) or clathrin heavy chain (CLTC), indicating its importance in the regulation of trafficking in the endolysosomal pathway [15].

The endosomal sorting complexes required for transport (ESCRT) are heteromeric protein complex composed of ESCRT-0, -I, -II, or -III. These complexes have been shown to regulate protein trafficking in the endolysosomal pathway and the fusion of autophagosomes with lysosomes in the autophagy pathway [16, 17]. Interestingly, mutations in CHMP2B, which is a major component of ESCRT-III, have been identified in chromosome 3-linked FTD (FTD-3), and the disease-associated CHMP2B^Intron5^ causes defects in protein trafficking in the endolysosomal pathway and in the accumulation of autophagosomes due to an impairment in the fusion of autophagosomes with lysosomes [18, 19]. It has also been shown that transgenic mice expressing FTD-3-linked CHMP2B^Intron5^ in their forebrain show defects in social behavior, which is a symptom exhibited by patients with FTLD [20]. CHMP2B has also been shown to regulate the maturation and maintenance of dendritic spines, synaptogenesis, and synaptic potentiation during chemical long-term potentiation [21, 22]. Intriguingly, both FTLD-associated CHMP2B and TMEM106B have been identified in the endolysosomal pathway, and both are known to regulate dendritic morphology in neurons [21, 23].

Although a number of recent studies have examined the cellular functions of TMEM106B in lysosomes, the function of TMEM106B in ESCRT-associated endosomal and autophagy pathways has not been elucidated. Furthermore, whether the increased expression of TMEM106B modulates CHMP2B^Intron5^-mediated endosomal defects and neurotoxicity is unknown. In this study, we examined whether TMEM106B variants were associated with the ESCRT pathway and whether disease-associated TMEM106B expression affected the cellular pathogenic effects of CHMP2B^Intron5^ in cultured cortical neurons. We found that the T185 variant associated more with CHMP2B and reduced autophagic flux compared to S185. Furthermore, T185 expression enhanced the CHMP2B^Intron5^-mediated endosomal/autophagic defects and neurotoxicity to a greater extent than S185. The findings of this study suggest that the T185 variant of TMEM106B is a risk factor for neurodegeneration associated with endosomal defects.

## Results

### Localization of endogenous TMEM106B to CHMP2B^WT^- or CHMP2B^Intron5^-positive structures in cultured cortical neurons

**Fig. 1:**
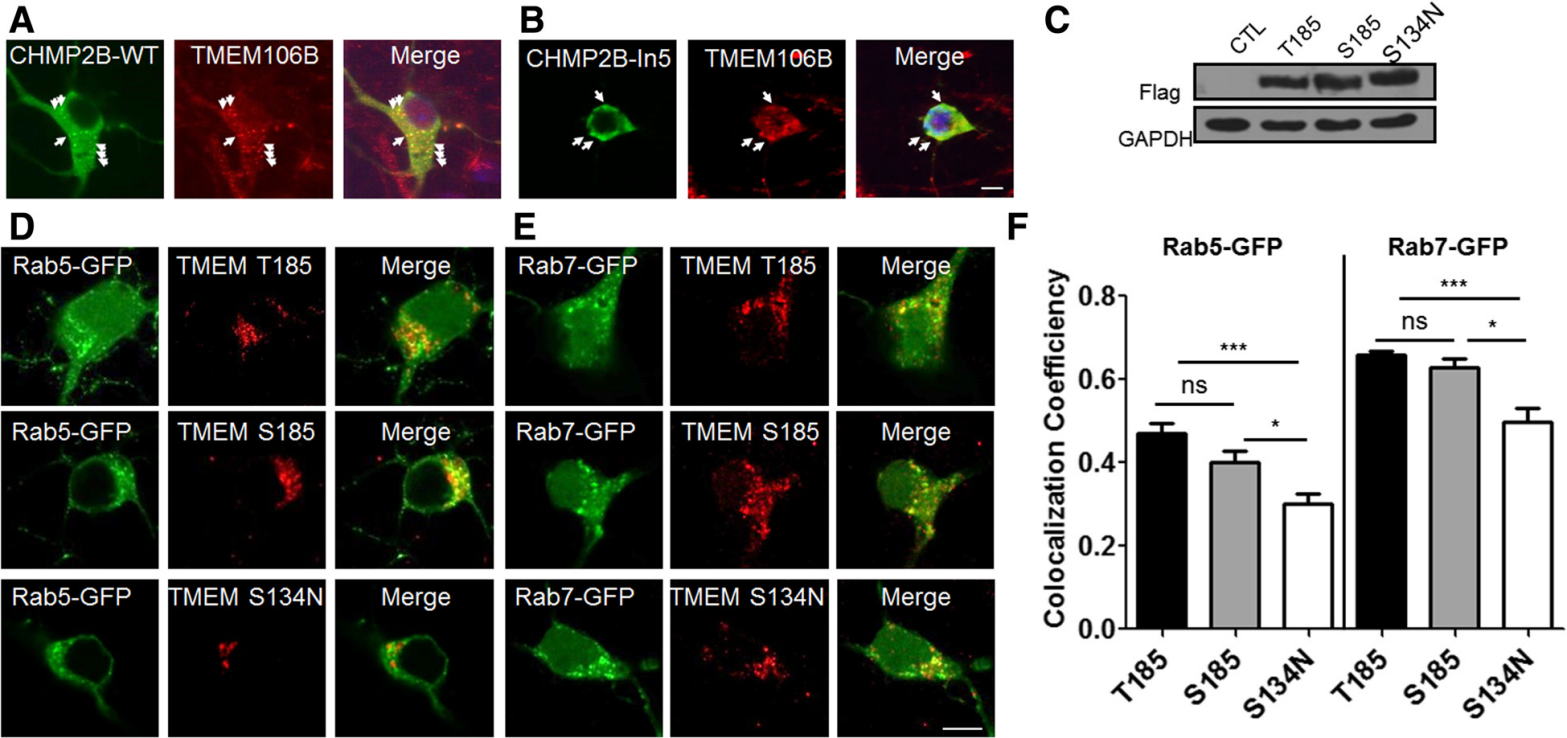
TMEM106B was localized to CHMP2B-positive structures, and the T185, S185, and S134N SNPs of TMEM106B were localized to Rab5- and Rab7-positive endosomes. **a** Confocal images showing the partial colocalization of endogenous transmembrane protein 106B (TMEM106B) and either charged multivesicular body protein 2B (CHMP2B)^WT^ or CHMP2B^Intron5^ (CHMP2B^In5^). Flag-tagged CHMP2B^WT^ and (**b**) flag-tagged CHMP2B^In5^ were expressed in cultured cortical neurons. Twenty-four hours after transfection, the cells were fixed and stained with anti-flag and anti-TMEM106B antibodies. The arrow indicates the colocalization of TMEM106B and CHMP2B. Scale bar: 10 μm. **c** Each flag-tagged TMEM106B single-nucleotide polymorphism (SNP; T185, S185, and S134N) was transfected into HEK293T cells. Twenty-four h after transfection, western blot analyses were performed with an anti-Flag or anti-glyceraldehyde 3-phosphate dehydrogenase (GAPDH) antibody. GAPDH was used as a loading control. Each flag-tagged TMEM106B SNP (T185, S185, and S134N) was cotransfected with either green fluorescent protein (GFP)-Rab5 (**d**) or GFP-Rab7 (**e**) in cultured cortical neurons. Twenty-four hours after transfection, the neurons were fixed and stained with an anti-flag antibody. **f** The coefficients of colocalization were measured and analyzed with the ImageJ program (Colocalization Indices). The values are presented as the mean ± standard error of the mean (SEM) of three independent replicates. One-way analysis of variance (ANOVA) followed by Tukey’s multiple comparisons test; *p < 0.05, ***p < 0.001; ns, not significant

In order to determine whether TMEM106B was involved in the ESCRT-associated endosomal pathway, we first examined the cellular localization of TMEM106B in CHMP2B^WT^***-*** and CHMP2B^Intron5^***-***expressing cortical neurons [23]. As shown in Fig. 1a, endogenous TMEM106B was partially colocalized to CHMP2B-positive endosomal structures. Interestingly, some TMEM106B-positive particles were adjacent to CHMP2B-positive structures, which indicated its possible association with the ESCRT complex in the endosomal pathway. In order to examine its association with CHMP2B in pathological conditions that are associated with the dysfunctional ESCRT complex, we investigated its localization in FTD-3-linked CHMP2B^Intron5^-expressing neurons [23, 24]. Endogenous TMEM106B was sequestered into abnormal CHMP2B^Intron5^ aggregates (Fig. 1b). Interestingly, CHMP2B^Intron5^-expressing neurons contained larger TMEM106B particles that were associated with CHMP2B^Intron5^-positive particles, which raised the possibility of a pathogenic effect of dysfunctional ESCRT-induced neurodegeneration.

### Greater localization of T185 and S185 to endosomal structures compared to S134N

The ESCRT complex is involved in the regulation of protein trafficking from early endosomes to late endosomes [25, 26]. If TMEM106B is involved in the ESCRT-associated endosomal pathway, it may be localized to Rab5- and Rab7-positive endosomes and function in association with the ESCRTs. We generated SNP variants of TMEM106B [p(T185, S185, and S134N], which have been identified in patients with FTLD, in order to better understand their functional significance and investigate differences in their cellular effects in endosomal pathways in the same cellular model system. In order to characterize each TMEM106B SNP variant in the endosomal pathway, the protein expression of each flag-tagged TMEM106B SNP (T185, S185, and S134N) was examined in HEK293T cells. Their levels of protein expression were similar 24 h after transfection in our cellular system (Fig. 1c). In order to examine their cellular localization in cortical neurons, each flag-tagged TMEM106B SNP was co-transfected with green fluorescent protein (GFP)-Rab5, an early endosomal marker, or GFP-Rab7, a late endosomal marker. As shown in Fig. 1d–f, all of the variants were more localized to Rab7-positive late endosomes compared to Rab5-positive early endosomes. However, the S134N variant was localized less to both early and late endosomes compared to the T185 and S185 variants, even though its levels of protein expression were similar to the levels of the other variants (Fig. 1b–c). This suggests that the T185 and S185 variants might affect the ESCRT-mediated endosomal pathway.

### Greater association of the T185 variant with CHMP2B compared to the S185 variant

**Fig. 2:**
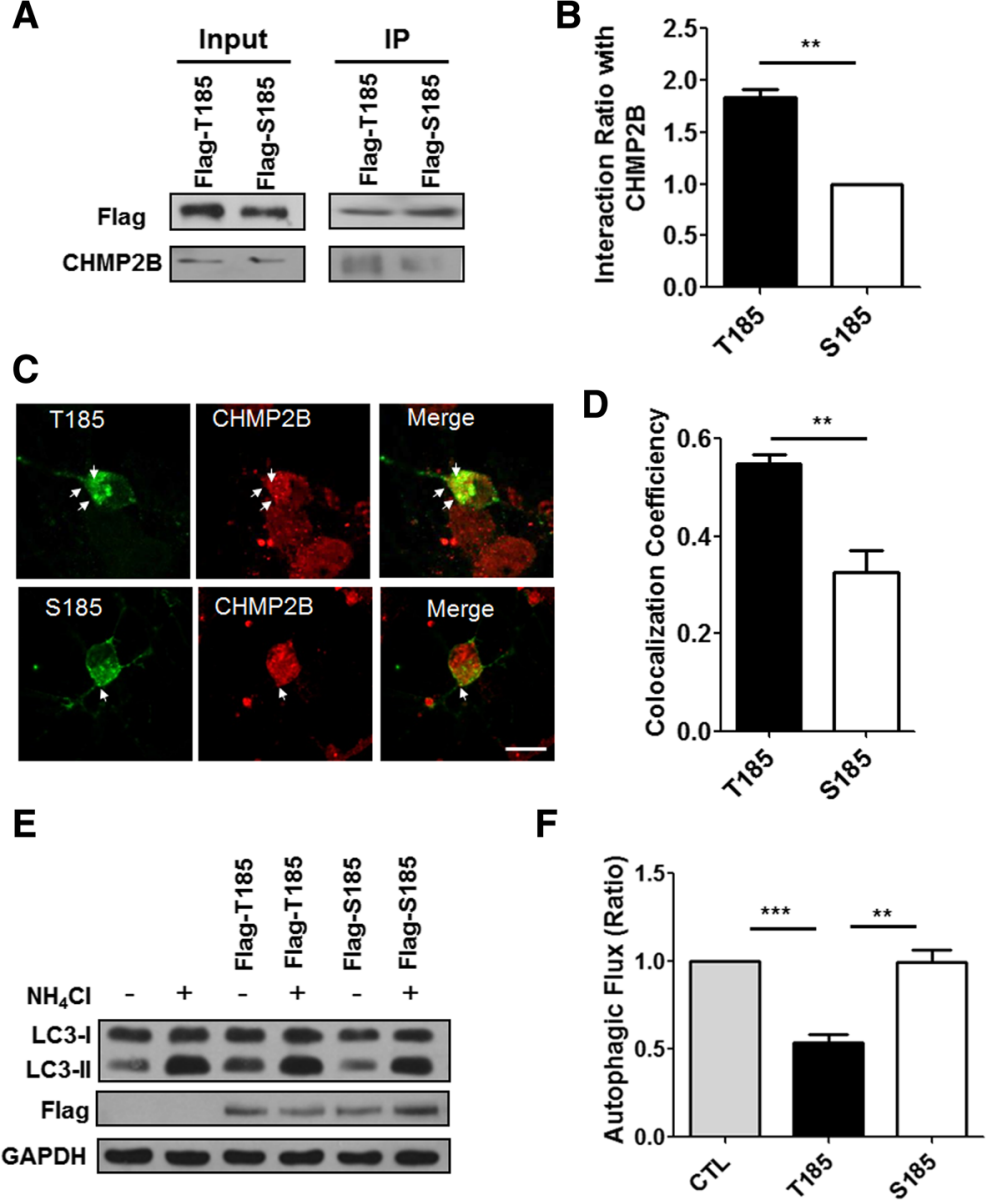
The T185 variant of TMEM106B was more associated with endogenous CHMP2B and reduced autophagic flux compared to the S185 variant. **a** Flag-tagged T185 or Flag-S185 was transfected into the HEK293T cells. Twenty-four to 48 h after transfection, a protein coimmunoprecipitation assay was performed with an anti-flag antibody. Western blot analyses were performed with an anti-CHMP2B or anti-flag antibody. **b** The bar graph represents the percentage of interaction of T185 or S185 with endogenous CHMP2B. For quantification, the band intensity of CHMP2B from the immunoprecipitation samples was normalized to that of either flag-T185 or flag-S185. The values are presented as the mean ± SEM of three independent replicates. Student’s *t*-test, **p < 0.01 **c** Each flag-tagged TMEM106B SNP (T185 or S185) was transfected into cultured cortical neurons. Twenty-four hours after transfection, the neurons were fixed and stained with an anti-flag antibody and anti-TMEM106B antibody. The arrows indicate the colocalization of CHMP2B with either T185 or S185. Scale bar: 20 μm. **d** The bar graph indicates the colocalization of endogenous CHMP2B with either T185 or S185 of TMEM106B. The coefficients of colocalization were measured and analyzed with the ImageJ program (Colocalization Indices). The values are presented as the mean ± SEM of three independent replicates. Student’s *t*-test; **p < 0.001. **e** Flag-tagged T185 or Flag-tagged S185 was transfected into HEK293T cells. Twenty-four h after transfection, the transfected or control cells were incubated with or without ammonium chloride (NH_4_Cl) for 24 h. The cell lysates were then subjected to western blot analyses with an anti-flag, anti-microtubule-associated protein 1A/1B-light chain 3 (LC3), or anti-GAPDH antibody. **f** The autophagic flux indicates the difference in the LC3-II levels in the presence and absence of NH_4_Cl. The autophagic flux ratio in the T185- or S185-expressing cells was normalized to that of the control cells. The values are presented as the mean ± SEM of three independent replicates. One-way ANOVA followed by Tukey’s multiple-comparisons test; **p < 0.01, ***p < 0.001

In order to distinguish the effects of T185 from S185 in ESCRT-associated endosomal pathways and autophagy pathways, we examined their association with CHMP2B, a component of the ESCRT-III complex that regulates protein trafficking and autophagic degradation [25]. Flag-tagged T185 and S185 were expressed in HEK293T cells. A protein co-immunoprecipitation analysis was performed with an anti-flag antibody, and a western blot analysis was performed with anti-CHMP2B and anti-flag antibodies. As shown in Fig. 2a–b, T185 and S185 were partially associated with endogenous CHMP2B. S185 was less associated with CHMP2B compared to T185 although protein stability is similar between T185 and S185 (Additional file 1: Figure S1). In order to further examine these associations in cortical neurons, we examined the cellular localization of T185 and S185 in cortical neurons expressing flag-tagged T185 or S185. The neurons expressing T185 or S185 were fixed and then stained with anti-flag and anti-CHMP2B antibodies. As shown in Fig. 2c–d, endogenous CHMP2B was partially localized with T185 and S185. Our colocalization analysis showed that T185 was associated more with CHMP2B, in line with our co-immunoprecipitation data (Fig. 2c–d).

### Autophagic flux was slightly reduced in cells expressing T185, but not in cells expressing S185

If TMEM106B associates with CHMP2B, what is its role in the ESCRT-involved endolysosomal pathway? Because TMEM106B associates with CHMP2B in endosomal pathways and functions in lysosomes, TMEM106B might be involved in autophagy with reference to its association with CHMP2B [27–29]. In order to investigate this question, we examined whether the expression of the TMEM106B variants affected autophagic flux. In order to measure autophagic flux, the levels of microtubule-associated protein 1A/1B-light chain 3 (LC3)-II in cells expressing T185 or S185 in the presence or absence of the lysosomotrophic reagent ammonium chloride (NH_4_Cl) were quantified in a western blot analysis [30, 31]. As shown in Fig. 2e–f, cells expressing T185 showed a slight reduction in autophagic flux compared to cells expressing S185. These results suggested that the enhanced sequestration of CHMP2B might reduce autophagic flux.

### T185 was more sequestered in CHMP2B^Intron5^-positive aggregates than S185

**Fig. 3:**
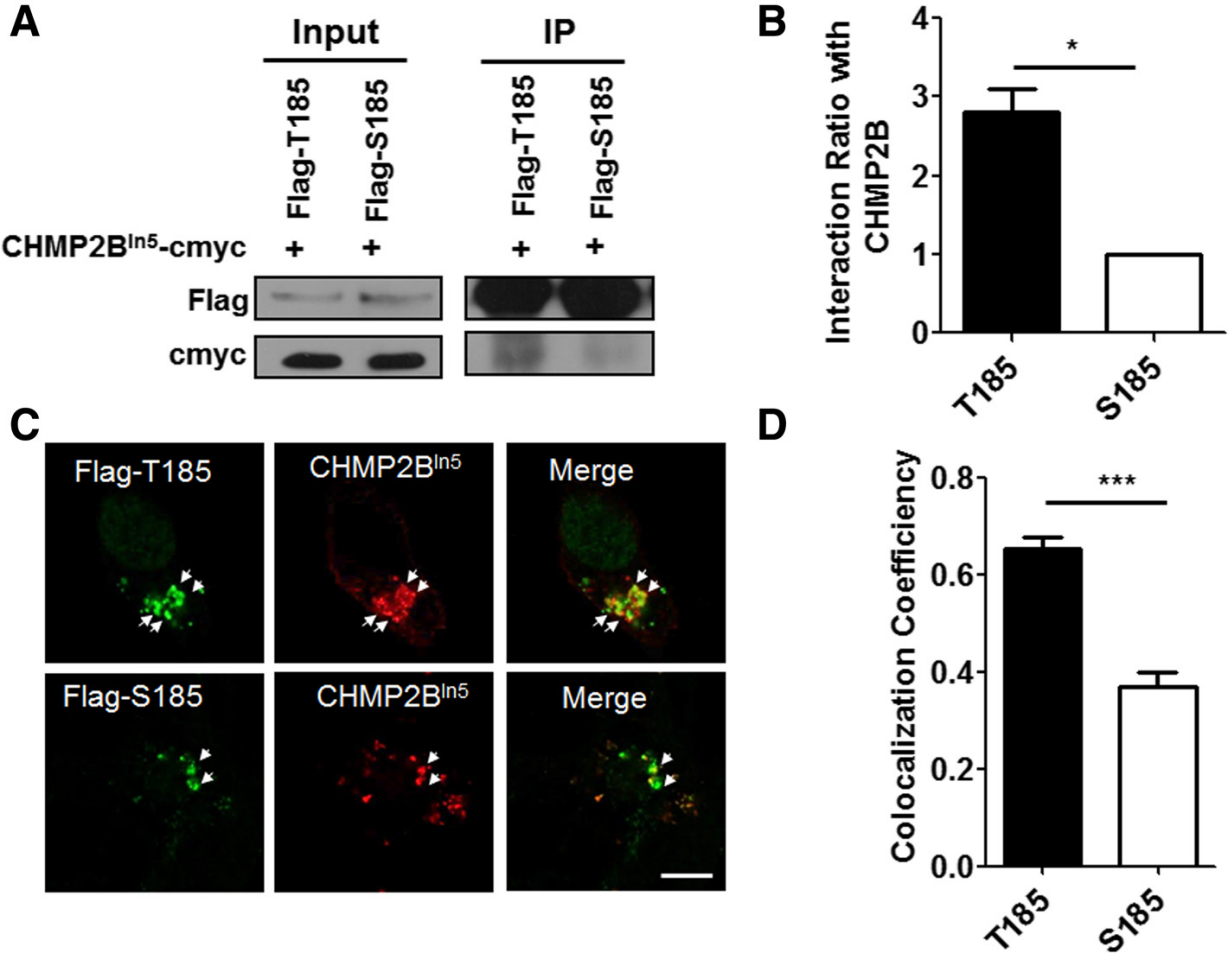
T185 was sequestered more in CHMP2B^Intron5^-positive aggregates. **a** CHMP2B^Intron5^-cMyc (CHMP2B^In5^-cMyc) was cotransfected with either Flag-tagged T185 or S185 into the HEK293T cells. Twenty-four to 48 h after transfection, the cell lysates were used for coimmunoprecipitation with an anti-flag antibody. Western blot analyses were performed with anti-cMyc or anti-flag antibodies. **b** The bar graph represents the percentages of interaction of T185 or S185 with CHMP2B^In5^. For quantification, the band intensity of CHMP2B^In5^ from the immunoprecipitation samples was normalized to that of either flag-tagged T185 or flag-S185. The values are presented as the mean ± SEM of three independent replicates. Student’s *t*-test, *p < 0.05. **c** Each flag-tagged TMEM106B SNP (T185 or S185) was cotransfected with CHMP2B^In5^-cMyc into cultured cortical neurons. Twenty-four hours after transfection, the neurons were fixed and stained with an anti-flag antibody and anti-cMyc antibody. The arrows indicate the colocalization of CHMP2B with either T185 or S185. Scale bar: 20 μm. **d** The bar graph indicates the colocalization of CHMP2B^In5^ with either the T185 or S185 isoform of TMEM106B. The coefficients of colocalization were measured and analyzed with the ImageJ program (Colocalization Indices). The values are presented as the mean ± SEM of three independent replicates. Student’s *t*-test; ***p < 0.001

Next, we examined the effects of diseases that are associated with TMEM106B as a risk factor in CHMP2B^Intron5^-mediated neurotoxicity. First, we examined the associations of T185 and S185 and the FTD-linked mutant CHMP2B^Intron5^. Flag-tagged T185 and S185 were transfected with CHMP2B^Intron5^-cMyc. Co-immunoprecipitation was performed with an anti-flag antibody, and a western blot analysis was done with an anti-cMyc antibody. As shown in Fig. 3a–b, the T185 variant was more associated with CHMP2B^Intron5^ than S185. We then examined the cellular localization of flag-tagged T185 and S185 in cortical neurons expressing CHMP2B^Intron5^. T185 was mostly sequestered into abnormal CHMP2B^Intron5^-positive aggregates, while S185 was partially sequestered into aggregates (Fig. 3c–d). These results suggested the involvement of T185 in the CHMP2B^Intron5^-mediated cellular defects and neurotoxicity.

### T185 slightly enhanced the CHMP2B^Intron5^-mediated endosomal/autophagic defects compared to S185

The results of our cellular study showed that, among the variants of TMEM106B, T185 was associated more with CHMP2B and reduced autophagic flux to a greater extent than S185 (Figs. 2 and 3). Therefore, we wanted to compare the effects of T185 and S185 on CHMP2B^Intron5^-mediated neurotoxicity. The expression of TMEM106B is increased in the brains of patients with FTLD [32, 33]. We next investigated whether T185 or S185 expression affected the neurotoxicity that is mediated by the FTD-3-linked mutant CHMP2B [19, 23]. Our previous report showed that the degradation of the epidermal growth factor receptor (EGFR) was delayed in CHMP2B^Intron5^-expressing cells [13, 19, 23].

**Fig. 4:**
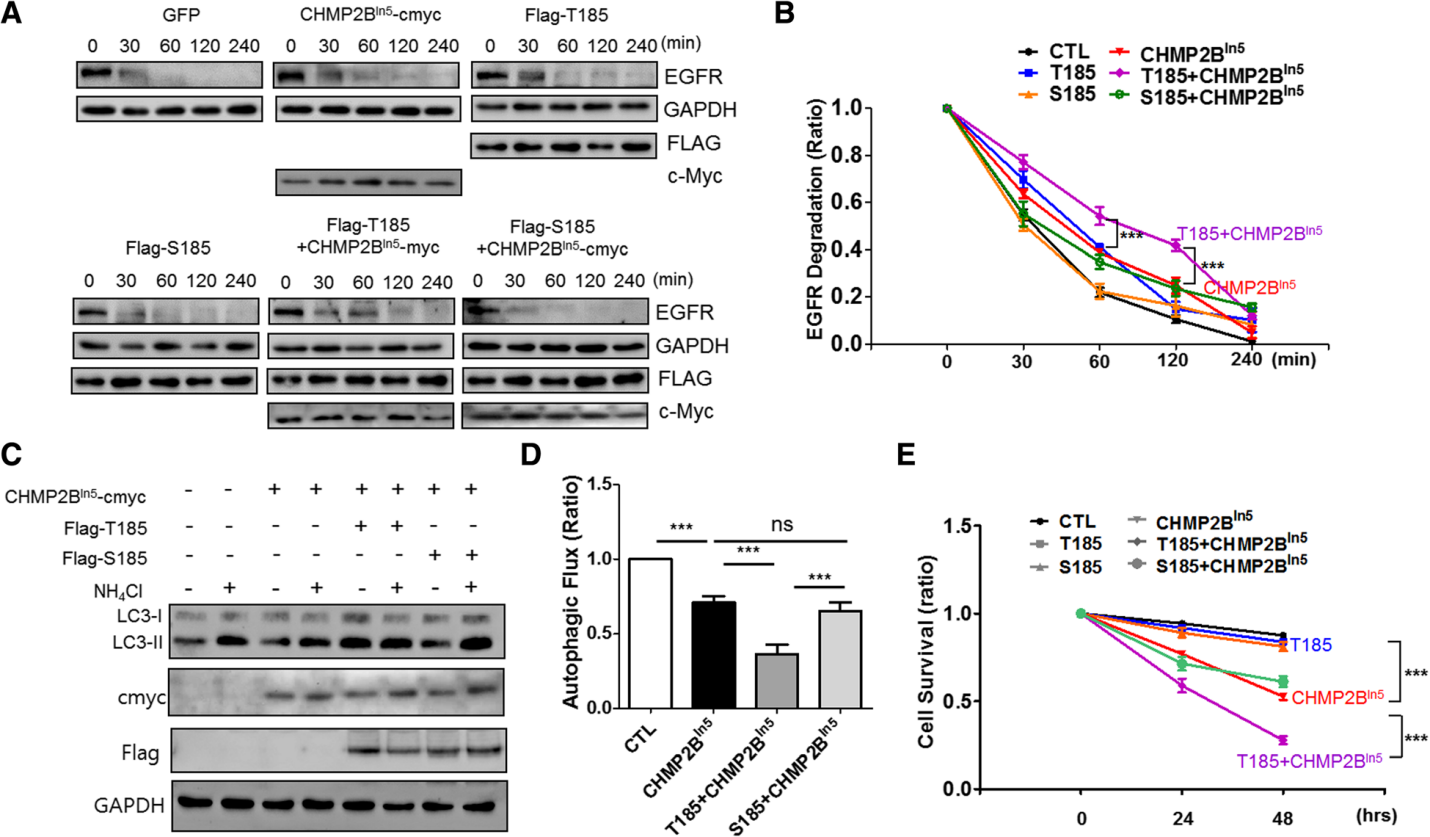
T185 slightly enhanced FTD-linked CHMP2B^Intron5^-mediated endo/autosomal defects and neurotoxicity, compared to S185. **a** GFP (CTL), Flag-tagged T185, Flag-tagged S185, and/or CHMP2B^In5^ were transfected into the HEK293T cells. Twenty-four to 48 h after transfection, the cells were incubated only with DMEM for 3 h and then treated with human epidermal growth factor (100 ng/mL) and cycloheximide (CHX, 30 μg/mL) for the indicated times (0, 30, 60, 120, or 240 min). Western blot analyses were conducted on the cell lysates with an anti-flag, anti-epidermal growth factor receptor (EGFR), anti-cMyc, or anti-GAPDH antibody. **b** The EGFR degradation was normalized to the levels of GAPDH. The graph represents the percentage of EGFR degradation that occurred during the indicated time. The percentage of EGFR degradation in each sample was normalized to that of EGFR in the control cells. The values are presented as the mean ± SEM of three independent replicates. One-way ANOVA followed by Tukey’s multiple-comparisons test; ***p < 0.001. **c** CHMP2B^In5^-cMyc was cotransfected with or without flag-tagged T185 or flag-tagged S185 into HEK293T cells. Twenty-four to 48 h after transfection, the transfected cells or control cells were incubated with ammonium chloride (NH_4_Cl) or without it for 24 h, and then the cell lysates were subjected to western blot analyses with an anti-flag, anti-cMyc, anti-LC3, or anti-GAPDH antibody. **d** Autophagic flux indicates the difference in the LC3-II levels in the presence and in the absence of NH_4_Cl. The autophagic flux in the CHMP2B^In5^-cMyc expressing cells with (or without) T185 or S185 expression was normalized to that of the control cells to represent autophagic ratio compared to the control cells. The values are presented as the mean ± SEM of three independent replicates. One-way ANOVA followed by Tukey’s multiple-comparisons test; ***p < 0.001. **e** Flag-tagged T185, flag-tagged S185 or/and CHMP2B^Intron5^ were cotransfected with GFP into cultured cortical neurons. The surviving neurons were counting the GFP-positive and propidium iodide (PI)-negative cells. The cell survival graph indicates the survival percentages of neurons expressing Flag-tagged T185 and/or CHMP2B^Intron5^-cMyc and GFP. The values are presented as the mean ± SEM of three independent replicates. One-way ANOVA followed by Tukey’s multiple-comparisons test; ***p < 0.001

In order to examine whether T185 or S185 affects the delayed degradation of the EGFR by CHMP2B^Intron5^, flag-tagged T185, flag-tagged S185, or an empty vector were coexpressed with CHMP2B^Intron5^-cMyc, and an EGFR degradation assay was performed. As reported previously, EGFR protein in CHMP2B^Intron5^-expressing cells remained at significant levels 30 and 60 min after EGF treatment (Fig. 4a–b) [23]. However, T185 further enhanced the accumulation of EGFR that was induced by CHMP2B^Intron5^ expression 30 and 120 min after EGF treatment (10 ng/mL), and the expression of T185 caused a slight delay in EGFR degradation compared to S185 (Fig. 4a–b). We next investigated whether T185 or S185 could modulate the reduction in autophagic flux that was caused by CHMP2B^Intron5^. The levels of LC-II in the presence or absence of NH_4_Cl in CHMP2B^Intron5^-cMyc-expressing cells with (or without) flag-tagged T185 or flag-tagged S185 were quantified in a western blot analysis. As shown in Fig. 4c–d, T185 amplified/enhanced the CHMP2B-induced reduction in autophagic flux to a greater degree than S185. These findings suggested that T185 enhanced the endosomal defects in receptor degradation and the autophagic flux defects caused by CHMP2B^Intron5^.

### T185 increased CHMP2B^Intron5^-mediated neurodegeneration

Although TMEM106B is considered a risk factor for FTLD-TDP and FTLD with *C9ORF72* expansions, little is known about its cellular pathogenic effects on CHMP2B^Intron5^-mediated neurodegeneration. Thus, to investigate whether T185 affected neuronal survival and CHMP2B^Intron5^-mediated neurotoxicity, flag-tagged T185 or flag-tagged S185 were co-transfected with GFP into mature cortical neurons, and the surviving neurons were counted 2–3 days later. As shown in Fig. 4e, T185 or S185 expression did not cause significant neuronal death 24–72 h after transfection in the cultured cortical neurons. However, T185, but not S185, significantly increased CHMP2B^Intron5^ expression-induced cell death. These results suggest that the T185 variant of TMEM106B functions as a risk factor that might enhance neuronal toxicity associated with neurodegeneration with endosomal defects, such as ESCRT dysfunction-associated neurodegeneration.

## Discussion

TMEM106B, which is localized to late endosomes and lysosomes, has been implicated in FTLD-TDP, which is associated with a *PGRN* mutation [7, 9], and its functions have been mainly characterized in lysosomes. Both TMEM106B and CHMP2B are localized to late endosomes and regulate neuronal morphology in polarized postmitotic neurons, suggesting their possible involvement in neuronal function [14, 21, 22]. However, little is known about their functional associations and the effects of diseases that involve TMEM106B in the ESCRT-mediated endosomal pathway and neurodegeneration with endosomal defects, such as ESCRT dysfunction. Here we present novel evidence of the association between TMEM106B and CHMP2B, and the effects of TMEM106B on CHMP2B^Intron5^-mediated endosomal/autophagic defects and neurotoxicity. Based on the results of our cellular studies, endogenous TMEM106B was associated with CHMP2B-positive endosomes and early/late endosomal structures. Moreover, endogenous TMEM106B was mostly sequestered into disease-mutant CHMP2B^Intron5^-positive aggregates in cultured cortical neurons. The results of this study indicated that TMEM106B was involved in the ESCRT-mediated pathway and that its functional loss through abnormal sequestration contributed to the disease phenotype associated with FTD-3-linked CHMP2B^Intron5^. A recent study showed that loss of TMEM106B reduced dendritic lysosomes and altered dendritic arborization [14]. Indeed, CHMP2B^Intron5^ expression causes dendritic retraction and defects in the maturation of dendritic spines, which suggests that it may be linked to TMEM106B in the regulation of dendritic morphology [21, 23]. Future detailed characterizations of the functional association of TMEM106B and CHMP2B in the regulation of dendritic morphology and other neuronal functions will be needed.

Moreover, we investigated the effects of SNP variants in the exon region of TMEM106B on the ESCRT-mediated pathway. Among the variants of TMEM106B, S134N was less localized to early/late endosomes compared to T185 or S185, which indicated that the T185 and S185 forms affect the ESCRT-mediated pathway. Although a previous *in silico* analysis predicted the reduced stability of S134N [7], our western blot analysis showed no significant differences in the protein expression levels of S134N and the other variants, at least in our cell culture model system. Detailed functional characterizations of the involvement of S134N in the cellular pathogenesis of FTLD are needed.

Our co-immunoprecipitation and colocalization analyses showed that T185 was associated more with CHMP2B and CHMP2B^Intron5^ compared to S185. This was unexpected because previous studies have reported that S185 degrades faster than T185, potentially due to differences in N-glycosylation at residue N183 [10]. However, 24–48 h after transfection in our cultured system, neither protein expression levels nor protein stability of T185 and S185 significantly differed from one another (Fig 1c, Additional file 1: Figure S1). Furthermore, the co-immunoprecipitation results clearly showed that CHMP2B associated less with the same amount of S185 compared with T185, which indicated that an amino-acid substitution (T- > S) might affect their protein structure and protein-protein interactions.

We also found that T185 expression reduced autophagic flux. The role of TMEM106B in the autophagy pathway remains unclear, although it is known that TMEM106B is localized to late endosomes/lysosomes. Indeed, it is well known that late endosomes can fuse with autophagosomes in autophagic maturation and that many lysosomal proteins and late endosomal proteins, such as ESCRT-III protein and Rab7 GTPase, are involved in this step of the autophagic process [28, 34–36]. Thus, the dynamic and transient interaction of the ESCRT complex, including the interaction of CHMP2B with TMEM106B, might affect the autophagic process. However, the greater association of T185 with CHMP2B might impair the normal steady state of the autophagic process compared to S185, which would result in reduced autophagic flux. Moreover, TMEM106B expression has been reported to cause the translocation of transcription factor EB, which controls autophagy and lysosomal biogenesis under stress, further implicating the possible link between TMEM106B and autophagy [15]. Future studies designed to elucidate the putative role of TMEM106B in autophagic regulation will provide additional insights into the mechanisms involved.

According to the results of our cellular studies in cultured cortical neurons, T185 enhanced CHMP2B^Intron5^ mutant-mediated endo/autosomal defects and neurotoxicity compared to S185, thus suggesting that TMEM106B might also be a risk factor in ESCRT-dysfunctional neurodegeneration. Increased levels and disordered expression patterns of TMEM106B have been reported in the brains of patients with FTLD [37]. More recently, TMEM106B has been reported as a genetic modifier of disease in carriers of *C9ORF72* expansions, which are also involved in the endolysosomal pathway [2, 4]. A growing body of evidence has shown that endolysosomal dysfunctions are closely associated with the cellular pathogenesis of several forms of FTLD [38]. Although TMEM106B was first identified in FTLD-TDP, SNPs in the coding region of TMEM106B might affect cellular signaling and protein-protein interactions that are involved in the endo-lysosomal pathway. Therefore, it is possible that TMEM106B might have more general effects on neurodegenerative diseases associated with defects of the endolysosomal pathway, such as Alzheimer’s disease and Parkinson’s disease, than previously believed. Future work employing transgenic mouse models or induced pluripotent stem cell-derived cellular models will be essential for revealing *in vivo* physiological and pathological functions of TMEM106B and its SNPs. The findings of the present study contribute to a better understanding of the role of TMEM106B in the cellular pathogenesis of neurodegenerative diseases that are associated with the endolysosomal and autophagy pathway and as a risk factor for these diseases.

## Conclusion

The results of this study provide novel evidence of an association between TMEM106B and the ESCRT-III complex protein, CHMP2B. In addition, we show that TMEM106B affects the ESCRT-mediated endosomal and autophagy pathways. Our functional characterization of the SNPs of TMEM106B showed that in comparison to the S185 isoform, the T185 isoform associated more with CHMP2B and reduced autophagic flux to a greater extent, thus enhancing the CHMP2B^Intron5^-induced endosomal/autophagic defects and neurodegeneration. Further characterization of TMEM106B in the ESCRT-mediated pathway and autophagy pathway may open up new avenues for the development of disease-modifying therapies in neurodegeneration that is associated with endolysosomal defects, as well as in FTLD.

## Methods

### DNA constructs

The human *TMEM106B* gene was cloned by nested polymerase chain reaction (PCR) from the cDNA of HeLa cells, and the *TMEM106B* mutations were then generated with recombinant PCR by using specific primer sets (Nested PCR primers: 1^st^ set, Forward: 5′-ccttgtcttaactacaaac-3′, Reverse: 5′-cattgagagtataggaaatatc-3′; 2^nd^ set, Forward: 5′-ctcctcagacatgggaaagtc-3′, Reverse: 5′-ctttaaatccatctcttccag-3′; T185, 5′-aaacaacataagcattattggt-3′, Reverse: 5′-accaataatgcttatgttgtt-3′; S134N, Forward: 5′-gcctatgtcaattatgatgtt-3′, Reverse: 5′-aacatcataattgacataggc-3′). For the mammalian expression, the DNA fragments were amplified with specific sets of primers (Forward: 5′-gaagatctggccaccatgggaaagtctctttct-3′ and Reverse: 5′-gctctagattactgttgtggctgaag -3′) and then subcloned into a 3XFLAG CMV 7.1 vector by using a BglII-XbaI site.

### Cell cultures and transfection

Primary cortical neurons were isolated from embryonic day 18 ICR mouse (Samtako Co., Ltd., Osan, Korea and DBL, Korea). HEK293T cells were grown in Dulbecco’s Modified Eagle’s Medium (DMEM) (Thermo Fisher Scientific Inc., Waltham, MA, USA) that was supplemented with 10 % fetal bovine serum (Thermo Fisher Scientific Inc.) and 1 % Penicillin-Streptomycin (Thermo Fisher Scientific Inc.) at 37 °C in 5 % CO_2_. Each plasmid DNA was transfected with Lipofectamine 2000 (Thermo Fisher Scientific Inc.) or Ca^2+^-phosphate reagents (Clontech Laboratories, Inc., Mountain View, CA, USA) in HEK293T cells or differentiated cortical neurons after 4–6 days *in vitro,* according to the manufacturer’s protocol.

### Immunocytochemistry, image analysis, and statistical analysis

Transfected cells were fixed with 4 % paraformaldehyde (Noblechem; #PAR500) for 10 min. For immunostaining, the transfected cells were permeabilized with 0.1 % Triton X-100 (Merck & Co., Inc., Kenilworth, NY, USA; #108603) and then blocked with 3 % bovine serum albumin (Sigma-Aldrich Co. LLC, St. Louis, MO, USA; #A7906) in phosphate-buffered serum (Welgene, Inc., Daegu, Korea) for 1 h at room temperature. The primary antibodies [anti-FLAG M2 (Sigma-Aldrich Co. LLC, F1804), anti-CHMP2B (Abcam PLC, Cambridge, UK; ab33174), anti-TMEM106B (Proteintech Group, Inc., Chicago, IL, USA; #20995-1-AP), anti-cMyc (EMD Millipore, Billerica, MA, USA; #06-549) and secondary antibodies (Jackson ImmunoResearch Laboratories, Inc., West Grove, PA, USA; #715-545-150, #715-165-150, #711-165-152, and #711-485-152) were incubated for 1 h each at room temperature. All of the photomicrographs were captured with a confocal microscope (Carl Zeiss AG, Jena, Germany; LSM 510). Image analysis and colocalization analyses were performed using ImageJ program (NIH) .

GraphPad Prism 5 was used for statistical analysis. The values are presented as the mean ± SEM of three independent replicates. One-way ANOVA followed by Tukey’s multiple-comparisons test for statistical analysis of more than three data sets or Student-t-test for statistical analysis of two groups was selected.

### Coimmunoprecipitation and western blotting

Forty-eight h after transfection with the plasmid DNA, the HEK293T cells were lysed with immunoprecipitation lysis buffer [50 mM of Tris-HCl (pH 7.5), 150 mM of NaCl, 1 % NP40, 0.5 % sodium deoxycholate, and protease inhibitor (F. Hoffmann-La Roche AG, Basel, Switzerland)]. The total cell lysates were incubated with 3 μg of the anti-FLAG antibody overnight at 4 °C and then incubated with Protein G agarose beads for 7 h at 4 °C. Subsequently, the protein complexes were washed with immunoprecipitation lysis buffer three times at 4 °C. For the western blotting, the samples were separated on 9–11 % sodium dodecyl sulfate-polyacrylamide gel electrophoresis gels and then transferred to polyvinylidene fluoride membranes (F. Hoffmann-La Roche AG). After blocking with 5 % nonfat milk, the membranes were incubated with the primary antibody and horseradish peroxidase-conjugated anti-mouse or anti-rabbit secondary antibody.

### Autophagic flux assay and quantification

To analyze autophagic flux, flag-tagged T185 or Flag-S185 was transfected with (or without) CHMP2B^In5^ into HEK293T cells. Twenty-four hours after transfection, the transfected or control cells were incubated with or without ammonium chloride (NH_4_Cl) for 24 h. The cell lysates were then subjected to western blot analyses with an anti-flag, anti-microtubule-associated protein 1A/1B-light chain 3 (LC3), or anti-GAPDH antibody. For quantification of autophagic flux, band intensity in each sample of western blot was measured using the ImageJ software. The difference in the LC3-II levels in the presence and absence of NH_4_Cl in each group was quantified from band intensity. Autophagic flux indicates the difference in the LC3-II levels in the presence and absence of NH_4_Cl. To compare the autophagic flux of each sample compared to control, we normalized the autophagic flux of each sample to that of the control cells (Autophagic flux ratio, control = 1). The values are presented as the mean ± SEM of three independent replicates.

### EGFR degradation assays and protein degradation assay

To assay EGFR degradation, 48 h after the transfection, the cells were starved in DMEM alone for 3 h. Human epidermal growth factor (100 ng/mL) and cycloheximde (30 μg/mL) were incubated for the indicated times (30, 60, 120, or 240 min). For the protein degradation assay, 24 h after transfection, the cells were washed with 1XPBS and cycloheximide (30 μg/mL) and were incubated for the indicated times (0, 24, or 48 h). The cell lysates that were prepared in RIPA buffer were subjected to western blotting with anti-EGFR, anti-FLAG, anti-MYC, or anti-glyceraldehyde 3-phosphate dehydrogenase (GAPDH) antibody and horseradish peroxidase-conjugated anti-mouse or -rabbit secondary antibody.

## Additional file

Additional file 1: Figure S1. (A) Flag-tagged T185 or flag-tagged S185 was transfected into HEK293T cells. Twenty-four hours after transfection, the cells were washed with 1XPBS and cycloheximide (CHX) (30 μg/mL) and incubated for the indicated times (0, 24, or 48 h). The cell lysates that were prepared in RIPA buffer were subjected to western blotting with anti-FLAG, or GAPDH antibody and horseradish peroxidase-conjugated anti-mouse or anti-rabbit secondary antibody. (B) Levels of EGFR was normalized to that of GAPDH for the indicated times (0, 24, or 48 h) in the presence of CHX. Bar graph represents the EGFR degradation ratio compared to the level of EGFR at 0 h after CHX treatment in T185 or S185 expressing cells. The values are presented as the mean ± SEM of three independent replicates. Student t-test; ns, not significant. (TIF 1336 kb)

## Abbreviations

*C9ORF72*: Chromosome 9 open reading frame 72
CHMP2B: Charged multivesicular body protein 2B
DMEM: Dulbecco’s Modified Eagle’s Medium
EGFR: Epidermal growth factor receptor
ESCRT: Endosomal sorting complex required for transport
FTD: Frontotemporal dementia
FTD-3: Chromosome 3-linked FTD
FTLD: Frontotemporal lobar degeneration
GFP: Green fluorescent protein
LC3: Microtubule-associated protein 1A/1B-light chain 3
PCR: Polymerase chain reaction
SNP: Single Nucleotide Polymorphism
TDP-43: TAR DNA-binding protein 43
TMEM106B: Transmembrane protein 106B
