# Supplementary figures and images for: TMEM106B, a frontotemporal lobar dementia (FTLD) modifier, associates with FTD-3-linked CHMP2B, a complex of ESCRT-III
Source: Mol Brain. 2015 Dec 10;8:85. doi: 10.1186/s13041-015-0177-z (PMC4676093; doi:10.1186/s13041-015-0177-z)

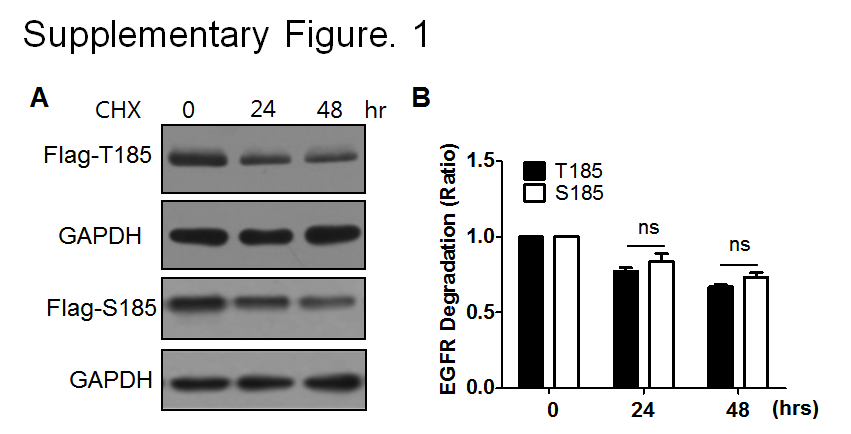

Supplement: Additional file 1: Figure S1. — (A) Flag-tagged T185 or flag-tagged S185 was transfected into HEK293T cells. Twenty-four hours after transfection, the cells were washed with 1XPBS and cycloheximide (CHX) (30 μg/mL) and incubated for the indicated times (0, 24, or 48 h). The cell lysates that were prepared in RIPA buffer were subjected to western blotting with anti-FLAG, or GAPDH antibody and horseradish peroxidase-conjugated anti-mouse or anti-rabbit secondary antibody. (B) Levels of EGFR was normalized to that of GAPDH for the indicated times (0, 24, or 48 h) in the presence of CHX. Bar graph represents the EGFR degradation ratio compared to the level of EGFR at 0 h after CHX treatment in T185 or S185 expressing cells. The values are presented as the mean ± SEM of three independent replicates. Student t-test; ns, not significant. (TIF 1336 kb) [file 13041_2015_177_MOESM1_ESM.tif]
